# Deepfake Detection Using the Rate of Change between Frames Based on Computer Vision

**DOI:** 10.3390/s21217367

**Published:** 2021-11-05

**Authors:** Gihun Lee, Mihui Kim

**Affiliations:** Department of Computer Science & Engineering, Computer System Institute, Hankyong National University, Jungang-ro, Anseong-si 17579, Gyeonggi-do, Korea; comb1001@hknu.ac.kr

**Keywords:** deepfake, computer vision, the rate of change

## Abstract

Recently, artificial intelligence has been successfully used in fields, such as computer vision, voice, and big data analysis. However, various problems, such as security, privacy, and ethics, also occur owing to the development of artificial intelligence. One such problem are deepfakes. Deepfake is a compound word for deep learning and fake. It refers to a fake video created using artificial intelligence technology or the production process itself. Deepfakes can be exploited for political abuse, pornography, and fake information. This paper proposes a method to determine integrity by analyzing the computer vision features of digital content. The proposed method extracts the rate of change in the computer vision features of adjacent frames and then checks whether the video is manipulated. The test demonstrated the highest detection rate of 97% compared to the existing method or machine learning method. It also maintained the highest detection rate of 96%, even for the test that manipulates the matrix of the image to avoid the convolutional neural network detection method.

## 1. Introduction

**Figure 1:**
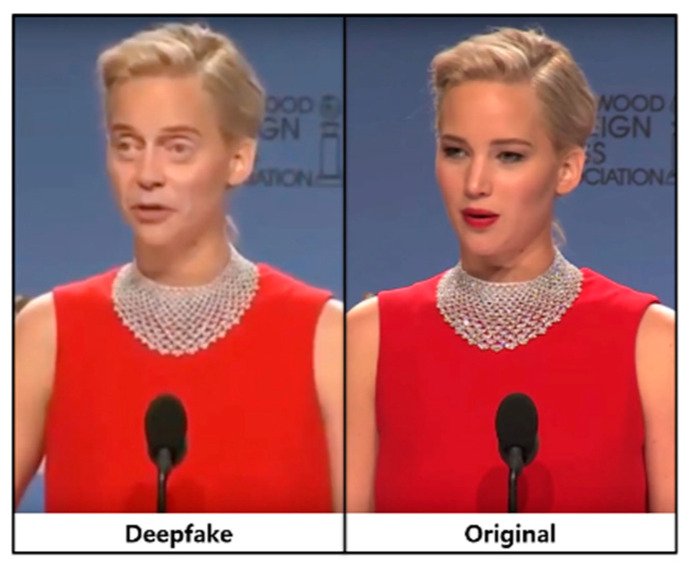
Deepfake image and original image [2].

Deepfake is a technology that uses artificial intelligence to synthesize another person’s face with the face of a person appearing in a video and manipulate the target person’s doing or saying things [1]. Deepfake technology has gradually developed and created videos that human eyes cannot distinguish (see Figure 1).

The development of deepfake technology poses a significant threat to digital content explosion owing to the development of smartphones and social networks. Particularly, problems include creating confusion in the stock market owing to false news, producing malicious effects on election campaigns, and generating regional political tensions between countries. Facial manipulation has been developed from modifying the lip motion of a person to synthesizing non-existent faces or manipulating the real face of one person [1]. Nowadays, Autoencoder and generative adversarial network (GAN) artificial intelligence have appeared. As a result, deepfake videos can be made easily for identity swapping.

Accordingly, various methods for detecting deepfakes have been proposed. Afchar et al. [3] proposed detection with a deep neural network using tiny noises in an image using convolutional neural network (CNN). Güera et al. [4] proposed detection using long short term memory (LSTM) by extracting features of the frame image of a video using a CNN. Li et al. [5] proposed extracting eye blinks using CNNs and detecting them using LSTM. Li et al. [6] proposed detection using the disparity of a distorted face using ResNet50 and the VGG16 model based on CNN. Yang et al. [7] proposed a method for extracting 68 landmarks from face images and detecting them using SVMs. Agarwal et al. [8] proposed detection using the dynamics of the mouth shape using a CNN. In most proposed methods, deepfakes are detected by extracting features from video frames using a CNN. However, CNNs are vulnerable to changes in metrics, such as blur, brightness, contrast, noise, and angle. Because a CNN has a convolutional filter of a specific size to extract features while moving around the image, if factors, such as blur, brightness, contrast, noise, and angle, change, differences from previously learned features occur. Test data with these changed factors have a lower detection rate in the learned CNN [9]. Therefore, in this study, computer vision features were extracted from the frames of videos without using a CNN, and then the rate of change of features between frames was calculated. We propose a method to detect deepfakes using the distribution of the data. The proposed method can detect manipulated digital content irrespective of changes in factors, such as blur, brightness, contrast, noise, and angle. In addition, a CNN must learn additional learning data by creating images with changed angles or contrasts to increase the detection rate. However, the proposed method can minimize these costs. Conversely, a CNN can detect manipulated digital content by extracting features from a single image, but the proposed method requires more than a certain number of frames to determine.

The contribution of this work is summarized as follows: First, we propose a method detecting deepfake video without a convolutional neural network. Usually, CNN learns a representation by embedding a vector in a hypersphere from an image. Then, it is used as the classifier’s input. In contrast, we extracted computer vision features first and used just a fully connected layer for classification. Second, we focus on detecting deepfake videos. Autoencoder and GAN make deepfake videos by manipulating frame by frame. We used unnatural differences between frames that can be made during manipulating. Thus, we calculated the rate of change between frames and used this for detecting deepfake videos. Third, we have many benefits because we do not use CNN. We can have comparable performance without data augmentation. Moreover, training time is saved because of the smaller parameter of the network and smaller datasets. Most importantly, our method is robust in regards to adversarial attacks or CNN’s weakness.

The remainder of this paper is organized as follows. Section 2 introduces the deepfake technology and existing deepfake detection methods. Section 3 describes the proposed deepfake detection method. Section 4 shows the feasibility of the proposed method by evaluating its performance and comparing it with other mechanisms.

## 2. Related Works

### 2.1. Deepfake Creation

**Figure 2:**
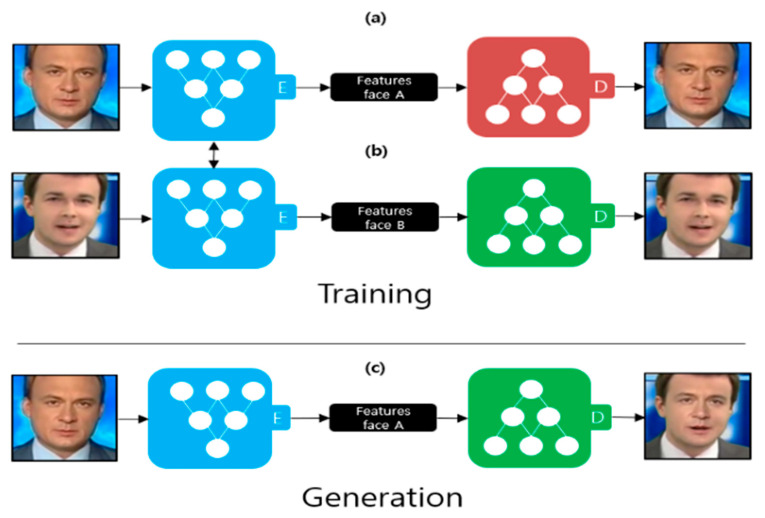
Deepfake creation process using an autoencoder [2]. (**a**) Autoencoder trained by face A; (**b**) Autoencoder trained by face B; (**c**) Deepfake creation process.

Deepfake is a technology that synthesizes the face of a character in a video into the face of a specific target using artificial intelligence technology. The artificial intelligence technologies used are primarily autoencoders [10] and the generative adversarial network [11]. Figure 2 illustrates the deepfake creation process using an autoencoder. An autoencoder comprises an encoder and a decoder. The goal of the encoder is to extract features from the image through dimensional reduction, and the goal of the decoder is to restore the original image as much as possible using the extracted features. Two autoencoders are used for learning to create a deepfake. The encoders, shown in Figure 2a,b, are trained using the same encoder. Therefore, the encoder learns common features that appear in face A (Figure 2a) and face B (Figure 2b). Examples of features include the position of the eyes, nose, and mouth. The decoders, depicted in Figure 2a,b, are trained separately. Figure 2c illustrates the deepfake creation process. After extracting the features of face A using an encoder, an image is generated using what the decoder learned, as shown in Figure 2b. FaceApp [2] is an example of deepfake production using an autoencoder.

**Figure 3:**
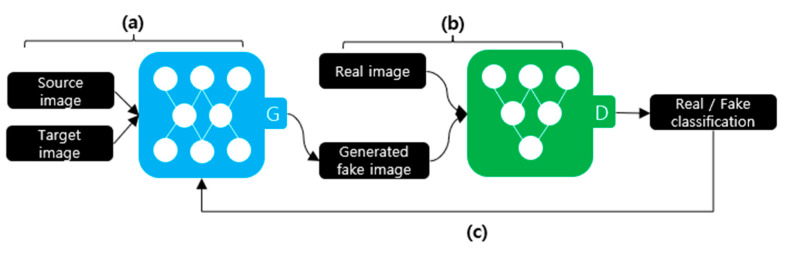
Deepfake creation process using a GAN. (**a**) Training the generator; (**b**) Training the discriminator; (**c**) Repeat.

Figure 3 illustrates the deepfake creation process using a GAN. A GAN comprises a discriminator and a generator. The generator, as depicted in Figure 3a, receives the source and target images to be synthesized as the input data. The generator creates a new image using the input data. The discriminator, as shown in Figure 3b, learns to distinguish between the real and generated fake images. As depicted in Figure 3c, this process repeats until the discriminator cannot distinguish between the generated fake image and the original image. StarGAN is an example of creating a deepfake using a GAN [12].

### 2.2. Deepfake Detections

Table 1 summarizes the methods proposed for deepfake detection in the past three years. Each proposed method can be classified as a key feature and architecture.

Afchar et al. [3] extracted features by analyzing mesoscopic noise from a single image using a CNN and then detected deepfakes using this feature. Microscopic analyses based on image noise cannot be applied in a compressed video context in which the image noise is strongly degraded.

Güera et al. [4] used a CNN and LSTM. The CNN extracts a feature vector of 2048 dimensions in units of frames. The LSTM receives the feature vector and detects the deepfake by searching for features with temporal significance between multiple frames.

Li et al. [5] used a CNN and LSTM. The CNN extracts the blinking patterns of the eyes. Using these extracted features, LSTM detects deepfakes by determining features with temporal significance between frames. The synthesized fake videos did not efficiently exhibit a physiological signal.

Li et al. [6] used VGG16 and ResNet50 models. These two neural networks are CNN-based neural networks. The CNN extracts the landmarks of the face to compute the transform matrices to align the faces to a standard configuration. The deepfake is detected by comparing the inconsistencies in the generated face areas and their surrounding regions. When creating a deepfake, matrix transformation occurs because limited images are used.

Yang et al. [7] used a CNN and an RNN. The CNN extracts features from each frame. The RNN detects the inconsistencies between frames from the extracted features. When creating a deepfake, inconsistencies may occur between frames because images are synthesized in units of frames.

Agarwal et al. [8] used a CNN. The CNN focuses on the visemes associated with words having the sound M, B, and P, in which the mouth must completely close to pronounce these phonemes. Deepfakes are detected using the inconsistencies between what is actually said and the shape of the mouth. Manipulated videos are occasionally inconsistent with spoken phonemes.

**Figure 4:**
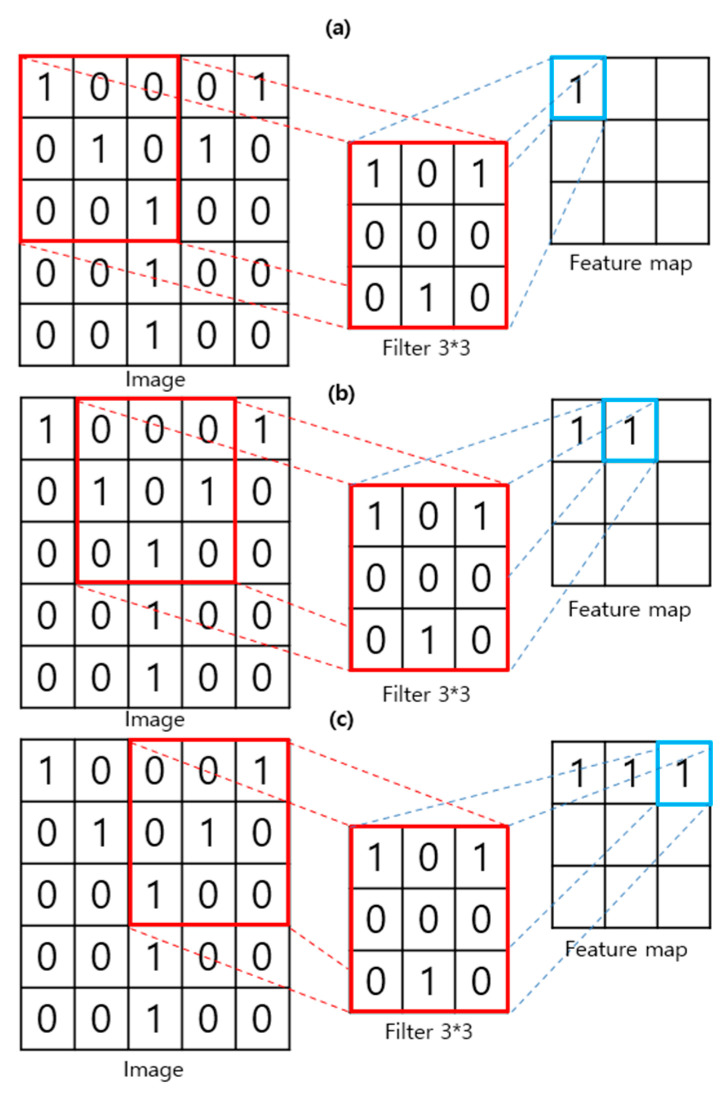
Feature map extraction process using a convolutional filter. (**a**) First step for feature extraction; (**b**) Next step (stride 1); (**c**) Next step (stride 1).

The deepfake detection methods proposed for the past three years detect deep fakes using a CNN. A CNN is a model that exhibits high performance, particularly related to image recognition, among artificial intelligence technologies [9]. Figure 4 illustrates a convolutional filter process that moves around the image by one space to create a feature map of the image. The convolutional filter is the core of CNN. This process results in the locality of pixel dependencies. It efficiently determines the small features of the image [13].

However, the performance is highly dependent on several factors in the image. When metrics, such as blur, brightness, contrast, noise, and angle, change, the detection rate of CNN drops significantly [9]. Malicious users can use this problem. Usually, an artificial network is trained by a dataset that has general representations. Malicious users could put just one filter to control with uncommon conditions in video. The eye can not feel the difference in people, but the pretrained network model cannot work properly in this image. In contrast, our method extracts computer vision features. Extracted features will change obviously. Nevertheless, our method focuses on the rate of change between frames. Each frame has the same condition change. Therefore, it is not critical for our method. These benefits make our method more robust in regards to CNN problems and adversarial attacks. Moreover, our model can be trained faster in a DFDC dataset that considers different acquisition scenarios, light conditions, distance from the camera, and pose variation. 

**Figure 5:**
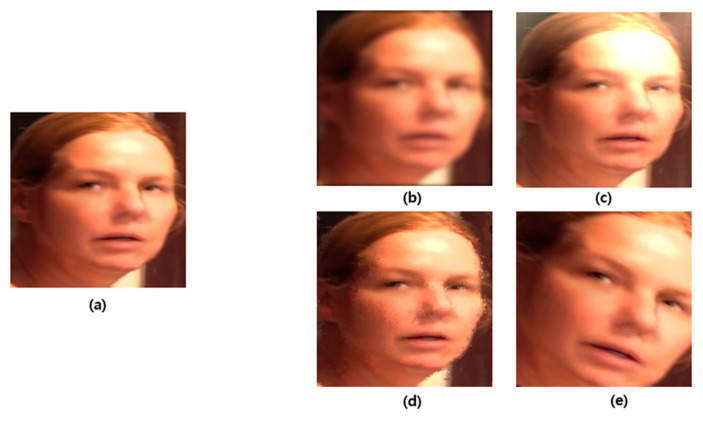
Example of undetectable image [15]. (**a**) Detectable deepfake image; (**b**) Undetectable deepfake image owing to Gaussian noise; (**c**) Undetectable deepfake image owing to brightness change; (**d**) Undetectable deepfake image owing to salt and pepper noise; (**e**) Undetectable deepfake image owing to angle change.

Figure 5 demonstrates an example in which the deepfake detection model using a CNN cannot detect. Figure 5a shows an image that can be detected as the frame of a general manipulated video. However, the remaining samples were not detected. Figure 5b shows the application of Gaussian noise in the manipulated frame. Figure 5c depicts changes in the brightness in the manipulated frame. Figure 5d shows the application of salt and pepper noise in the manipulated frame. Figure 5e depicts changes in the angle in the manipulated frame. The disadvantage of being undetectable owing to such a change in metrics can be used to avoid the CNN-based detection method [9,14].

## 3. Proposed System

**Figure 6:**
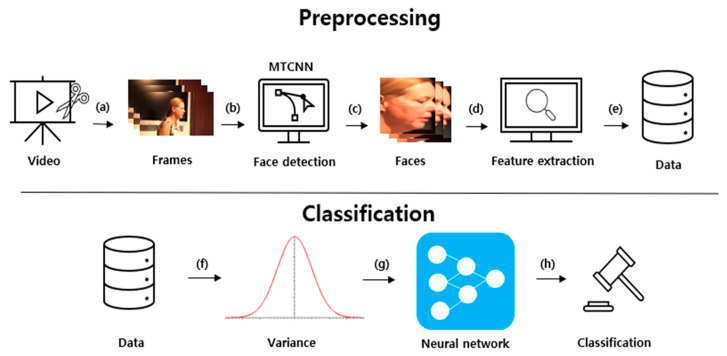
Proposed system structure [15]. (**a**) Extracting frames from video; (**b**) Face detection using MTCNN from each frames; (**c**) Crop detected faces; (**d**) Feature extraction from cropped faces; (**e**) Collecting extracted features; (**f**) Calculate variance from data; (**g**) Using neural network with data; (**h**) Classification from neural network.

Figure 6 demonstrates the proposed system structure. The method is divided into preprocessing and classification processes. The preprocessing process extracts a face image from a frame image, extracts computer vision features, and then extracts the difference between the frames. The classification process detects a deepfake using a DNN by obtaining the variance of a certain number of frames from the preprocessed data.

### 3.1. Preprocessing

First, the video was divided into frames, as shown in Figure 6a. Then, the face part was detected and cut using MTCNN [16] in each frame, as depicted in Figure 6b. MTCNN is a Python module that improves the accuracy of face detection by 95% accuracy compared to a CNN. By only extracting the face and measuring the amount of change, it can focus more on the transformation of the face in computer vision. The extracted face image frames were arranged, as demonstrated in Figure 6c. Subsequently, various computer vision features were extracted from the face image, as illustrated in Figure 6d. A feature vector was generated by extracting computer vision features from the aligned face images using computation, clustering, and filtering. 

**Figure 7:**
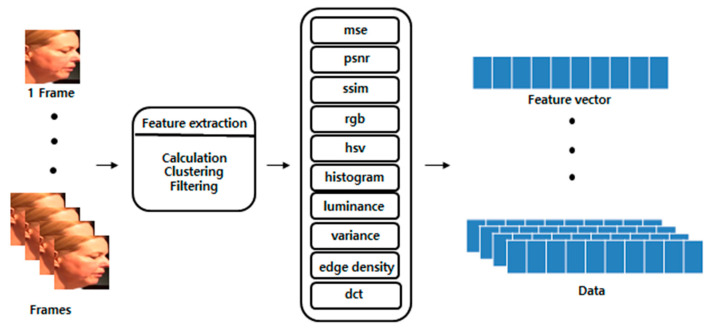
Computer vision feature extraction process [15].

The extracted features are presented in Table 2. The mean squared error (mse) measures the similarity of an image using the difference in the intensity of pixels between two images. The peak signal-to-noise ratio (psnr) evaluates the loss information for the image quality. psnr focuses on numerical differences rather than human visual differences. Because psnr is calculated using mse, when mse is 0, psnr is also set to 0. The structural similarity index measure (ssim) evaluates the temporal difference felt by humans in terms of luminance, contrast, and structural aspects. Red, green, blue (rgb), and the hue, saturation, and value (hsv) represent the color space of an image. The histogram represents the distribution of hues in the images. The luminance represents the average total brightness of the image. The variance represents the variance of the image brightness values. edge_density is the ratio of the edge components of all the pixels. The discrete cosine transform (dct) refers to the sharpness of an image. Because the deepfake production method synthesizes the target image for each frame, it may cause unnatural changes to various computer vision features. In addition, when creating a deepfake, the target image is obtained with limited resolution, and the size is changed as transformation matrices to fit the source image. Therefore, the sharpness is often inferior. In addition, distortion and blurring occur. The selected features greatly influence the deepfake creation process. Figure 7 demonstrates frames with a significant change rate value for each computer vision feature among data obtained by preprocessing from a single deepfake video. Figure 6e takes the absolute value after calculating the difference between the extracted computer vision features of the *i*-th frame from the *i* + 1-th frame. The degree of change in the computer vision features was different for each video. Therefore, the rate of change was calculated by dividing the change by the average value of the change between all video frames. 

Each feature is calculated using Equation (1). $f_{i}$ denotes the feature value of the *i*-th frame. $f_{i+1}$ denotes the feature value of the *i* + 1-th frame. mean(*f*) denotes the average of the feature values of all frames obtained from one video.

(1) $$f_{i}=\frac{\mathrm{abs}\left(f_{i+1}-f_{i}\right)}{mean\left(f\right)},$$

**Figure 8:**
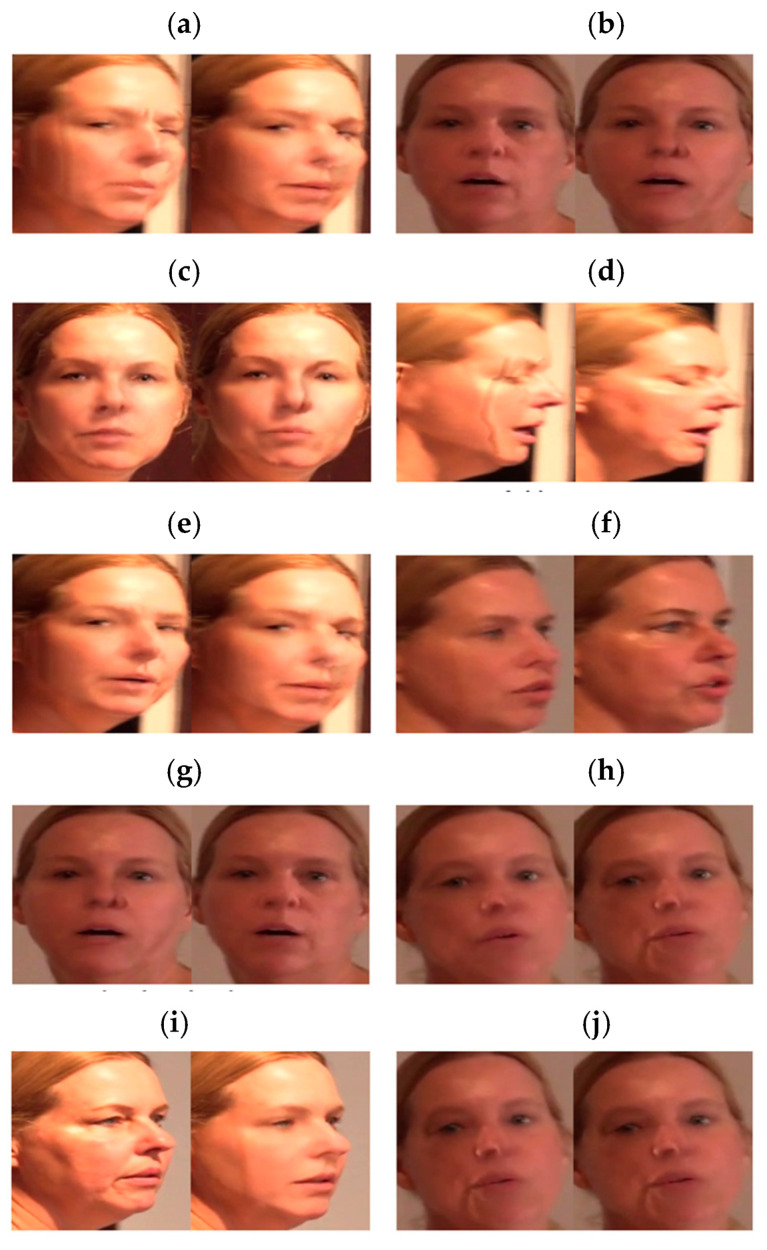
Frames showing a significant rate of change [15]. (**a**) mse; (**b**) psnr; (**c**) ssim; (**d**) rgb; (**e**) hsv; (**f**) histogram; (**g**) luminance; (**h**) variance; (**i**) edge density; (**j**) dct.

Figure 8 shows the extraction of the frame with the most significant change in each feature from one deepfake video.

### 3.2. Classification

The variance for each feature was calculated by grouping the rate of change between certain extracted frame numbers in Figure 6f. The calculated variance of each feature was used as the data for DNN learning. A dependent variable indicating whether the data is a deepfake video was attached. Finally, these data were learned by the DNN and used to detect deepfakes. The final data were calculated using Equation (2). $Data_{i}$ denotes one feature value of the i-th a data sample used for DNN learning. $d_{i}$ denotes the i-th data obtained by preprocessing.$\;\bar{d}$ denotes the average value of n data obtained by preprocessing.

(2) $$Data_{i}=\frac{1}{n}\sum_{i*n}^{n}{\left(d_{i}-\bar{d}\right)}^{2}$$

### 3.3. Modeling

Table 3 presents the accuracy by calculating the variance of the extracted adjacent frame change rate by a certain number. The highest accuracy of 95.22% was obtained when the DNN was trained by calculating the variance with 20 pieces of data.

Table 4 presents the accuracy by changing the optimizer function and the number of hidden layers to determine the appropriate hyperparameter. The Keras module was used for the learning. Image feature extraction was performed using OpenCV [17]. Binary cross-entropy was used as the loss function of the DNN. The highest accuracy of 97.39% was obtained when the DNN used the Adam optimizer function and five hidden layers.

When comparing our method and MesoNet using CNN, our model has 3–8 layers and has about 15,202 total parameters. On the other hand, MesoNet has 6–18 layers and has about 27,977 total parameters. Thus, our model has almost 50% fewer hyperparameters. Moreover, the training time is faster than Mesonet, by more than 30%, because it skips the data augmentation process.

## 4. Performance Evaluation

### 4.1. Dataset

A total of three datasets were used. The Face2Face and FaceSwap datasets are provided by FaceForensics++ [18]. This dataset contains more than 1000 videos. Kaggle provides the Deepfake Detection Challenge (DFDC) dataset [15]. This dataset is over 470 GB. The characters appearing in all datasets are composed of various races, genders, and various shooting environments. This study used 206 videos of Face2Face, 210 videos of FaceSwap, and 176 videos of DFDC for the experiment. Three hundred frames were extracted from one video, and the face size extracted using MTCNN was set to 160 × 160 pixels. Python 3 and the image processing library OpenCV were used to extract the computer vision features from each frame. To confirm the result was owing to the change in the metric in the frame, 15% of the frame images in the DFDC test dataset indicated a 10% metric change.

### 4.2. Evaluation

Each model was implemented in Python 3, and Keras was used for the machine learning model training. Table 5 lists the system specifications for the experiments. According to the dataset, the proposed methods, the Mesonet method using CNN, and the SVM method, were compared. Table 6 presents a comparison of the detection accuracy.

The Mesonet method using the Face2Face and FaceSwap datasets exhibited a higher than 90% detection accuracy. However, an experiment using the DFDC dataset with a changed metric showed a 77.71% detection accuracy. It could be inferred that the metric of the frame image was changed in the test data of the DFDC dataset, and the detection accuracy of the CNN was degraded.

The SVM [19] method for all datasets exhibited a detection accuracy of less than 60%. It could be inferred that detecting a deepfake video using only the rate of change between frames is difficult unless a major defect occurs when manipulating the image.

The proposed method using the Face2Face and FaceSwap datasets exhibited a detection accuracy of more than 95%. In addition, an experiment using the DFDC dataset with a changed metric exhibited 96.55% detection accuracy. Mesonet learned by creating a new image by changing metrics, such as the angle and contrast, of the training data. However, the proposed method exhibited a similar detection accuracy without additional learning. We used a similar amount of the dataset to other deepfake papers. However, the quality of the academic dataset is poor and not diverse. Therefore, if we use this method in a really good quality dataset, it will not be effective. Future studies will address these issues.

## 5. Conclusions

In this paper, to detect deepfake videos, we propose a method of extracting the rate of change of computer vision features between frames and using a DNN based on the variance of a certain number of frames. Unlike existing deepfake detection methods, the problem of avoiding detection methods owing to changes in various metrics was solved because a CNN was not used. In addition, the amount of training data was less than that of the existing CNN. The proposed method exhibited detection accuracies of 97.39% and 95.65% for the Face2face and FaceSwap datasets, respectively, and 96.55% for the DFDC dataset with the metric changed dataset.

## Figures and Tables

**Table 1:** Deepfake detection method proposed in the past three years.

Methods	Key Features	Architecture	Published
Microscopic analyses [3]	Mesoscopic properties of images	MesoNet (based on CNN)	2018
Temporal inconsistencies [4]	Frame level temporal features	CNN + LSTM	2018
Eye blinking [5]	Temporal patterns of eye blinking	CNN + LSTM	2018
Face warping [6]	Inconsistencies in warped face and surrounding area	VGG16, ResNet50 (based on CNN)	2019
Discrepancy [7]	Temporal discrepancies across frames	CNN + RNN	2019
Spoken phoneme mismatches [8]	Mismatches between the dynamics of the mouth shape	CNN	2020

**Table 2:** Extracted computer vision features.

Attribute	Explanation
mse	The average squared difference between the estimated values and the actual value
psnr	The ratio between the maximum possible power of a signal and the power of corrupting noise
ssim	The perceived quality of digital television and cinematic pictures
rgb	The percentage of each red, green, and blue color of the image
hsv	The percentage of each hue, saturation, and value of the image
histogram	The histogram plots the number of pixels in the image with a particular brightness or tonal value
luminance	The mean of the total brightness of the image
variance	Image variance of the image
edge_density	The ratio of edge pixels to the total pixels of in the image
dct	DCT bias of the image

**Table 3:** Accuracy by the number of distributed data.

Count	Accuracy
5	90.78%
10	92.33%
20	95.22%
30	86.67%
50	76.67%

**Table 4:** Hyperparameters—model performance.

Optimizer	# Hidden Layers	Loss	Accuracy
SGD	3	0.5560	67.83
	5	0.4146	78.26
	8	0.3439	81.74%
AdaGrad	3	0.6577	60.43%
	5	0.6672	55.22%
	8	0.6494	62.83
Adam	3	0.1608	94.35%
	5	0.0722	97.39%
	8	0.1120	94.78%

**Table 5:** System specification for the experiment.

CPU	AMD Ryzen 7 3800X 8-Core Processor
RAM	32 GB DDR4
GPU	Nvidia GeForce GTX 1660 Ti
VRAM	6 GB GDDR6

**Table 6:** Deepfake detection performance comparison.

	Face2face	FaceSwap	DFDC
**Proposed model**	97.39%	95.65%	96.55%
**Mesonet**	93.21%	95.32%	77.71%
**SVM**	54.24%	53.46%	52.91%

## References

[1] Ruben T., Ruben V.R., Julian F., Aythami M., Javier O.G. (2020). DeepFakes and Beyond: A Survey of Face Manipulation and Fake Detection. arXiv.

[2] Faceswap. https://faceswap.dev.

[3] Afchar D., Nozick V., Yamagishi J., Echizen I. MesoNet: A Compact Facial Video Forgery Detection Network. Proceedings of the 2018 IEEE International Workshop on Information Forensics and Security (WIFS).

[4] Güera D., Delp E.J. Deepfake Video Detection Using Recurrent Neural Networks. Proceedings of the 2018 15th IEEE International Conference on Advanced Video and Signal Based Surveillance (AVSS).

[5] Li Y., Chang M.-C., Lyu S. In Ictu Oculi: Exposing AI Created Fake Videos by Detecting Eye Blinking. Proceedings of the 2018 IEEE International Workshop on Information Forensics and Security (WIFS).

[6] Li Y., Lyu S. (2019). Exposing DeepFake Videos by Detecting Face Warping Artifacts. arXiv.

[7] Yang X., Li Y., Lyu S. (2018). Exposing Deep Fakes Using Inconsistent Head Poses. arXiv.

[8] Agarwal S., Farid H., Fried O., Agrawala M. Detecting Deep-Fake Videos from Phoneme-Viseme Mismatches. Proceedings of the 2020 IEEE/CVF Conference on Computer Vision and Pattern Recognition Workshops (CVPRW).

[9] Grm K., Štruc V., Artiges A., Caron M., Ekenel H.K. (2018). Strengths and weaknesses of deep learning models for face recognition against image degradations. IET Biom..

[10] Hou X., Shen L., Sun K., Qiu G. Deep Feature Consistent Variational Autoencoder. Proceedings of the 2017 IEEE Winter Conference on Applications of Computer Vision (WACV).

[11] Goodfellow I.J., Pouget-Abadie J., Mirza M., Xu B., Warde-Farley D., Ozair S., Courville A., Bengio Y. (2014). Generative Adversarial Networks. arXiv.

[12] Choi Y., Choi M., Kim M., Ha J.-W., Kim S., Choo J. StarGAN: Unified Generative Adversarial Networks for Multi-Domain Image-to-Image Translation. Proceedings of the 2018 IEEE/CVF Conference on Computer Vision and Pattern Recognition.

[13] Krizhevsky A., Sutskever I., Hinton G.E. (2017). ImageNet classification with deep convolutional neural networks. Commun. ACM.

[14] Roy P., Ghosh S., Bhattacharya S., Pal U. (2019). Effects of Degradations on Deep Neural Network Architectures. arXiv.

[15] Deepfake Detection Challenge|Kaggle. https://www.kaggle.com/c/deepfake-detection-challenge.

[16] Zhang K., Zhang Z., Li Z., Qiao Y. (2016). Joint face detection and alignment using multi-task cascaded convolutional networks. IEEE Signal Process. Lett..

[17] OpenCV. https://opencv.org/.

[18] Rössler A., Cozzolino D., Verdoliva L., Riess C., Thies J., Nießner M. (2019). FaceForensics++: Learning to Detect Manipulated Facial Images. arXiv.

[19] Schuldt C., Laptev I., Caputo B. Recognizing Human Actions: A Local SVM Approach. Proceedings of the 17th International Conference on Pattern Recognition.

